# Host-Specific Functional Significance of *Caenorhabditis* Gut Commensals

**DOI:** 10.3389/fmicb.2016.01622

**Published:** 2016-10-17

**Authors:** Maureen Berg, Xiao Ying Zhou, Michael Shapira

**Affiliations:** ^1^Department of Integrative Biology, University of California, BerkeleyBerkeley, CA, USA; ^2^Graduate Group in Microbiology, University of California, BerkeleyBerkeley, CA, USA

**Keywords:** *Caenorhabditis*, microbiota, microbiome, *Enterococcus faecalis*, host-microbe interactions, *Enterobacter cloacae*, hologenome, adaptation

## Abstract

The gut microbiota is an important contributor to host health and fitness. Given its importance, microbiota composition should not be left to chance. However, what determines this composition is far from clear, with results supporting contributions of both environmental factors and host genetics. To gauge the relative contributions of host genetics and environment, specifically the microbial diversity, we characterized the gut microbiotas of *Caenorhabditis* species spanning 200–300 million years of evolution, and raised on different composted soil environments. Comparisons were based on 16S rDNA deep sequencing data, as well as on functional evaluation of gut isolates. Worm microbiotas were distinct from those in their respective soil environment, and included bacteria previously identified as part of the *C. elegans* core microbiota. Microbiotas differed between experiments initiated with different soil communities, but within each experiment, worm microbiotas clustered according to host identity, demonstrating a dominant contribution of environmental diversity, but also a significant contribution of host genetics. The dominance of environmental contributions hindered identification of host-associated microbial taxa from 16S data. Characterization of gut isolates from *C. elegans* and *C. briggsae*, focusing on the core family *Enterobacteriaceae*, were also unable to expose phylogenetic distinctions between microbiotas of the two species. However, functional evaluation of the isolates revealed host-specific contributions, wherein gut commensals protected their own host from infection, but not a non-host. Identification of commensal host-specificity at the functional level, otherwise overlooked in standard sequence-based analyses, suggests that the contribution of host genetics to shaping of gut microbiotas may be greater than previously realized.

## Introduction

The gut microbiota is an important contributor to host health and fitness, impacting all aspects of life, from development, and metabolism to immunity and behavior (Shin et al., 2011; Levy et al., 2015; Sampson and Mazmanian, 2015; Sison-Mangus et al., 2015). Given its importance, it is expected that microbiota composition should not be left to chance. Traits enabling colonization by beneficial microbes would increase individual fitness, and thus be positively selected, giving rise to species-specific microbiotas shaped largely by host genetics. Support for these predictions includes the identification of species-specific microbiotas in various organisms (Moeller et al., 2014; Otani et al., 2014; Moran, 2015; Berg et al., 2016), demonstrations of host specificity among vertebrate gut commensals (Rawls et al., 2006; Frese et al., 2011), alignment of host phylogeny, and the composition of the associated microbiotas (Brucker and Bordenstein, 2012; Brune and Dietrich, 2015), and the heritability of certain gut microbiota taxa in twins (Goodrich et al., 2014, 2016). At the same time, evidence also indicates dominant contributions of environmental factors, including geographical diversity and diet, to shaping of the gut microbiota (Yatsunenko et al., 2012; Carmody et al., 2015). Shaping by the environment can allow microbiotas to respond to changing conditions, and increase host fitness. Thus, to which extent host genetics affects microbiota composition, and whether its effects are combined with those of the environment in complex gene-environment interactions is still not well understood. Here we describe a study using the *Caenorhabditis elegans* model and several of its related species aimed at gauging relative contributions of host genetics and the environment, focusing on environmental microbial diversity.

Recent work established *C. elegans* as a model for studying host-microbiota interactions (Berg et al., 2016; Dirksen et al., 2016). This popular genetic model has been used extensively for studying host-microbe interactions (Tan and Shapira, 2011; Gusarov et al., 2013; Meisel and Kim, 2014; Dierking et al., 2016). However, decades-long lab cultivation on monoxenic bacterial cultures has left us with very little knowledge about its natural history, including its interactions with natural commensals. This is beginning to change (Montalvo-Katz et al., 2013; Frézal and Félix, 2015; Berg et al., 2016; Dirksen et al., 2016; Samuel et al., 2016). Previous work in our lab established an experimental pipeline in which genetically-homogenous populations of initially germ-free worm larvae are grown to adulthood in composted soil environments, emulating habitats from which *C. elegans* has been isolated in the past. Using this pipeline, we employed 16S rDNA deep sequencing to characterize the gut microbiota in *C. elegans* of the standard N2 lab strain. This analysis revealed that worms raised in different microbial environments assembled distinct gut microbiotas that were more similar among worms from different environments than to microbiotas from the respective soil environment (Berg et al., 2016). Furthermore, changes in ambient temperature corresponded with small changes in worm microbiotas, which were distinct in their trends from those observed for the same taxa in the environment. Together, these results suggested that host factors—perhaps its physiology, and perhaps its genetics, were important in shaping the gut microbiota.

The experiments presented here examine the contribution of host genetics to microbiota composition, by identifying differences in microbiotas assembled in worms of different genotypes spanning 200–300 million years of nematode evolution (Pires-daSilva and Sommer, 2004). Microbiota characterization by deep sequencing identified host-specific patterns, and demonstrated a significant contribution of host genetics to microbiota composition. However, experimental variables (mainly differences between environmental microbiotas) affected worm microbiota composition more than the worm genotype. Effects of this environmental variability also hindered the identification of host-specific taxa. Attempts to overcome this by increasing taxonomical resolution and focusing on culturable bacteria of the *Enterobacteriaceae* core microbiota family, isolated either from *C. elegans* or from *C. briggsae*, similarly did not identify phylogenetic distinctions between commensals of the two species. However, functional evaluation of these gut isolates revealed host-adaptation in the form of host-specific contributions to development, infection resistance, and lifespan. These results support the role of host genetics in shaping microbiota composition, and suggest that the extent of this contribution may surpass what could be deduced based on the commonly available phylogenetic resolution.

## Materials and methods

### Strains

*C. elegans* wildtype strains included N2, Hawaiian (CB4856), and CB4857. Other species included *C. briggsae* (AF16), *C. tropicalis* (JU1373), and *Pristionchus pacificus* (PS312). All were obtained from the *Caenorhabditis* Genetic Center (CGC). Bacterial strains used were *E. coli* OP50-1, the Gram-positive pathogen *Enterococcus faecalis* (strain V583), the Gram-negative pathogen *P. aeruginosa* PA14, two previously isolated gut bacteria from *C. elegans, Pseudomonas mendocina* (Montalvo-Katz et al., 2013) and *Enterobacter cloacae* (Berg et al., 2016), and two additional *E. cloacae* strains isolated from *C. briggsae* in the current study.

### Soils

Soil near a eucalyptus tree supplemented with chopped over-ripe bananas was used in experiment 1. Soil near an olive tree supplemented with chopped apples, was used for the second. Soil-produce mixtures were allowed to decompose for 1–2 weeks in the lab prior to the addition of worms, as previously described (Berg et al., 2016).

### Worm growth and harvesting

Initially germ-free L1 larvae, obtained following bleaching of gravid worms to release eggs, and hatching on standard nematode growth medium (NGM) plates without food, were transferred to soil and grown at 25°C for 3 days. In a given experiment, one batch of prepared soil was split into separate 50-mL conical tubes (5 gr per vial), and independent worm populations were raised in each (three biological replicates per worm strain or species). Approximately 100 gravid worms were harvested from each population using a Baermann funnel lined with two layers of tissue paper, washed extensively, and surface sterilized prior to DNA extraction (or bacterial isolation), as described elsewhere (Berg et al., 2016).

### DNA isolation and sequencing library preparation

DNA isolation and sequencing library preparation were carried out as previously described, using nested PCR with barcoded primers amplifying the 16S V4 variable region (Delgado et al., 2013; Tremblay et al., 2015; Berg et al., 2016). Paired-end sequencing was performed either with an Illumina HiSeq 2,500 at Berkeley's Coates Genomics Sequence Laboratory (experiment 1), or with an Illumina MiSeq machine at the UC Davis Genome Center (experiment 2). Previous work demonstrated that results obtained with these two platforms were consistent (Caporaso et al., 2012)

### Sequence reads processing

V4 16S rDNA reads were quality-filtered using QIIME (v.1.9.0) with default parameters (Caporaso et al., 2010). Unlike the 250-bp paired-end reads generated by MiSeq, the 150-bp HiSeq paired-end reads did not overlap and could not be used together. Therefore, to equalize analysis of data from both experiments, only forward reads were used in downstream analyses (but see Figures S1, S3 and Table S2 for comparisons between results obtained from experiment 2 data based either on single-end or paired-end MiSeq reads, which demonstrated comparable taxonomic resolution and similar results). The longer MiSeq forward reads were not trimmed, as processing of full or trimmed sequences led to almost identical OTU tables with 99% overlap. In both experiments, around 90% of reads passed quality filtering, with an average of 150,000 reads per sample in experiment 1, and an average of 80,000 reads per sample in experiment 2. Filtered reads were clustered into OTUs at a 97% similarity cut-off using *uclust* (Edgar, 2010), and OTU tables were rarefied to 20,000 sequences per sample. Taxonomy was assigned to OTUs using the 13_5 Greengenes release, as previously described (Yatsunenko et al., 2012).

### 16S data analysis

Faith's phylogenetic diversity index was used as a measure of community (α) diversity, calculated based on the Greengenes reference tree (Faith, 1992).

Dissimilarity between microbiotas (beta diversity) was evaluated based on weighted UniFrac distances of rarefied counts, using Student's *t*-test, and assigning significance in comparison to a distribution of random scores generated by 1,000 Monte Carlo permutations of the UniFrac distance data.

Canonical Analysis of Principal Coordinates (CAP) was used to cluster microbiotas, based on weighted UniFrac distances of rarefied counts, in a multi-dimensional space constrained by both experiment and genotype variables. CAP was performed using the function capscale in the *vegan* R package (Oksanen et al., 2015). Significance was assessed using PERMANOVA.

Bacterial taxa characteristic of microbiotas from each genotype were identified using the R package *indicspecies* (De Cáceres and Legendre, 2009), as previously described (Berg et al., 2016). This analysis assesses the strength of the relationship between OTU abundance and different host genotypes by comparing OTU abundance in microbiotas of one genotype to their abundance in other genotypes. Enrichment values were calculated for each indicator as a log-transformed ratio of the abundance in worms over the abundance in soil.

### Isolation and identification of gut bacteria

Bacteria were isolated from worms grown in soils similar to those used for sequencing, and harvested as described above. Washed and surface-sterilized worms were ground using a motorized pestle in 300 μl of M9, pelleted, and bacteria from supernatant grown on plates with *Enterobacteriaceae*-selective medium (Violet Red Bile Glucose (VRBG), 25°C, 2 days). Isolates were identified through sequencing of the full-length 16S rDNA gene, amplified using primers 27f and 1492r (94°C-45 s, 50°C-60 s, 68°C-90 s; 40 cycles), and through multi-locus sequencing (MLS) of hsp60, gyrB, rpoB, [95°C-30 s, 50°C-30 s (60°C for hsp60), 72°C-45 s, 30 cycles, Paauw et al., 2008].

### Colony forming unit (CFU) counts

Around 100 harvested worms in a volume of 300 μL M9 salt solution were ground using a motorized pestle. Serial dilutions were plated on VRBG agar, and incubated for 24 h at 37°C before counting. Prior wash solution was also spread onto LB plates without antibiotics to verify lack of contaminating surface bacteria.

### Rate of development

Approximately 30 gravid worms were transferred to NGM plates pre-seeded with 200 μL of 10x concentrated overnight bacterial cultures, and allowed to lay eggs for 1 h. The number of progeny at each developmental stage was subsequently scored after 42 h.

### Survival assays

Survival assays were performed in triplicate with approximately 100 worms per group, per experiment. PA14 infection assays were performed on slow killing plates as previously described (Shapira and Tan, 2008), and *E. faecalis* infection assays were performed on Brain Heart Infusion Agar (Sifri et al., 2002). For infection assays, worms were transferred to the pathogen as L4 worms. Lifespan assays were performed on NGM plates, as described elsewhere (Twumasi-Boateng et al., 2012). All experiments were carried out at 25°C. Kaplan-Meier analysis was employed for statistical evaluation, followed by a log rank test.

### Data availability

Raw data can be downloaded from http://metagenomics.anl.gov/ (ref. number: 18910). OTU tables are provided in Table S1. Sequences used in identification of bacterial isolates were submitted to GenBank (accession numbers KX711976-KX712069).

## Results

### Host genotype and microbiota composition

To investigate the effect of host genotype on microbiota composition, worms of seven different genotypes were grown in soil enriched with rotting produce. Aliquots of one batch of soil were used for growing all genotypes, which included three wildtype strains of *C. elegans*-N2, CB4853 (CB), and Hawaiian (HI), and its relative species *C. briggsae* (Br), *C. tropicalis* (Tr), the gonochoristic *C. remenai* (Re), and the parasitic nematode *Pristionchus pacificus* (Ps) as an outgroup. Using a recently established experimental pipeline (Berg et al., 2016), initially germ-free L1 larvae of the examined strains were added to soil, grown to adulthood, and harvested for bacterial 16S rDNA deep sequencing. The procedure includes extensive washing and surface sterilization, and enables focusing on live gut bacteria, while demonstrating high reproducibility between technical replicates (Berg et al., 2016). One soil sample and 19 worm populations, including 2–3 independent populations for each genotype, were sequenced, generating around 150,000 sequences per sample. Sequences were clustered at a 97% sequence identity threshold, and sorted into 6,684 operational taxonomic units (OTUs, 2,026 of which identified only in soil, Table S1). Raw data can be downloaded from http://metagenomics.anl.gov/ (ref. number: 18910).

**Figure 1:**
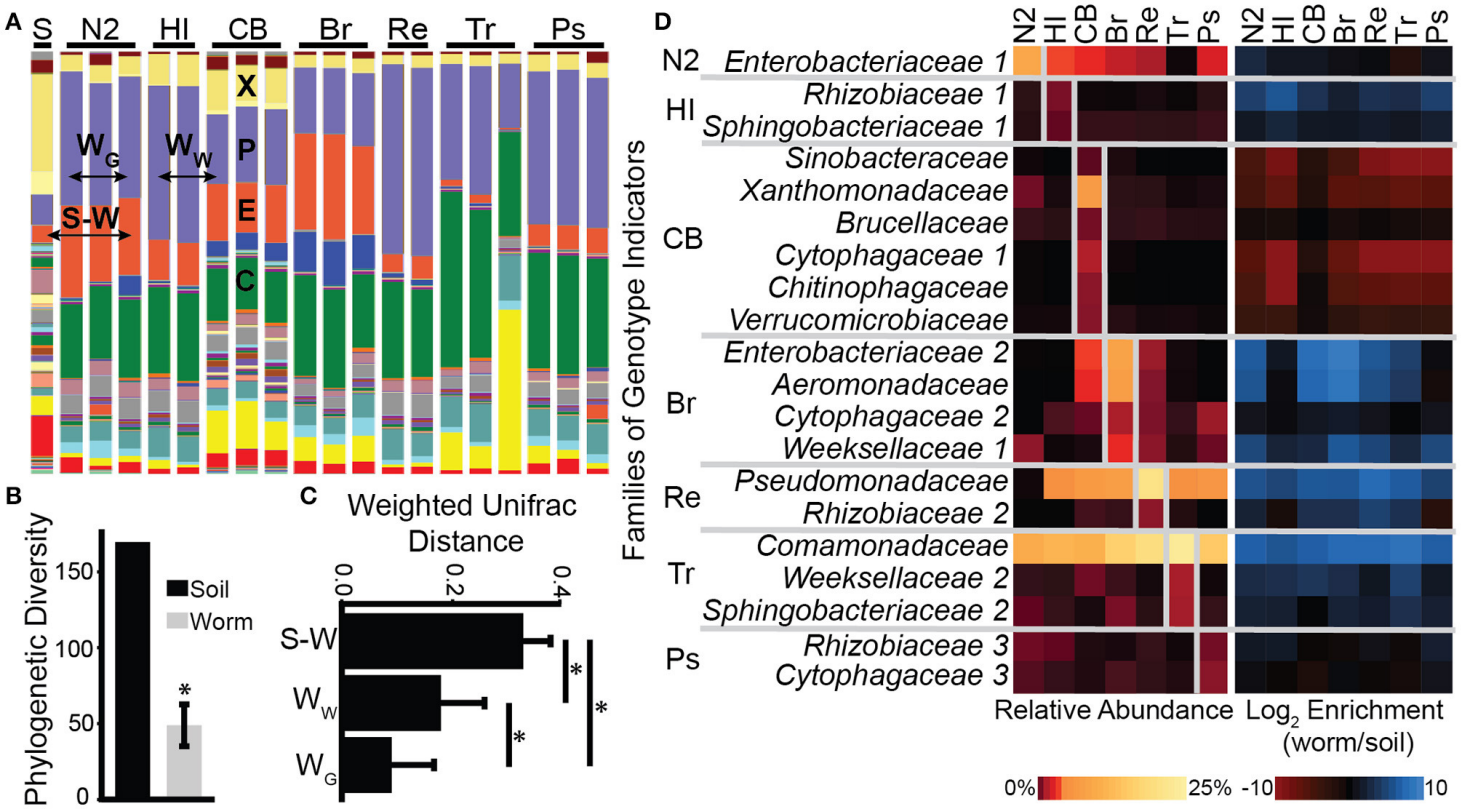
**Worm microbiota composition reflects differences in host genotype. (A)** Soil and worm microbiota composition. Each bar represents a microbiota from a worm population (>100 worms), or from their soil environment (S, 1 g), showing relative abundance of taxa (family-level, color-labeled). Worms include *C. elegans* strains N2, Hawaiian (HI), and CB4857 (CB), *C. briggsae* (Br), *C. remanei* (Re), *C. tropicalis* (Tr), and *Pristionchus pacificus* (Ps). Highlighted major families include *Enterobacteriaceae* (E), *Xanthomonadaceae* (X), *Pseudomonadaceae* (P), and *Comamonadaceae* (C). **(B)** Worm microbiotas are less diverse than the soil microbiota. Shown is microbial diversity in soil versus averages ± SDs for 19 worm microbiotas. ^*^p < 0.001 (Student's *t*-test). **(C)** Weighted UniFrac distances between microbiotas demonstrate greater similarity in worms of the same genotype (W_G_), than in worms of different genotypes (W_W_), and further greater than similarity between worm microbiotas and the soil microbiota (S-W); averages ± SDs for all possible pair-wise comparisons; ^*^p < 0.001 (Student's *t*-test with 1,000 Monte Carlo permutations). **(D)** Heat map of abundance (left) and enrichment compared to soil (right) of taxa differentiating between microbiotas from different host genotypes, with unique indicator OTUs pooled into the family level, and numbered to differentiate between OTU subsets within the same family. Only the most abundant families (>0.1% in any microbiota; see Table S3 for full list) are presented; shown are averages of triplicate measurements for each genotype, except HI and Re, with *N* = 2.

As observed before, worm microbiotas were distinct from the microbiota in their soil environment (Figure 1A, Berg et al., 2016). They were significantly less diverse (Figure 1B), and more similar in composition to other worm microbiotas than to the environmental microbial community from which they have originated (Figure 1C). Moreover, worm microbiotas of the same strain or species were more similar in their composition to each other than to those of other genotypes.

Overall, worm microbiotas were dominated by members of the *Enterobacteriaceae* (11 ± 8%), *Pseudomonadaceae* (28 ± 10%), *Xanthomonadaceae* (4 ± 2%), and *Comamonadaceae* (22 ± 8%) families. A previous characterization of the N2 *C. elegans* microbiota identified members of the first three families as core taxa. On the other hand, members of *Comamonadaceae* were not found to be typical of all worm microbiotas, but rather indicative of one of two identified worm microbiota subtypes (Berg et al., 2016). Whether the current worm microbiotas should be considered representatives of this particular subtype, or alternatively, whether *Comamonadaceae* should be considered a core taxa, cannot be resolved based on available data.

Aiming to identify genotype-specific commensals, we used the program *indicspecies* to identify taxa that were more likely to be found in one genotype than in others. Identified genotype-specific OTUs were non-overlapping, but in some cases were members of similar bacterial families (Figure 1D, Table S3). Thus, certain *Enterobacteriaceae* OTUs were found to be indicators of N2 microbiotas, while others were indicators of *C. briggsae* microbiotas. For the most part, indicator species were enriched in worms compared to soil (Figure 1D right panel). The exception was CB4857's indicators, whose abundance in CB4857 worms was similar to that in soil, but very much unlike their near exclusion in all other strains.

### Environmental diversity and host genotype shape microbiota composition

**Figure 2:**
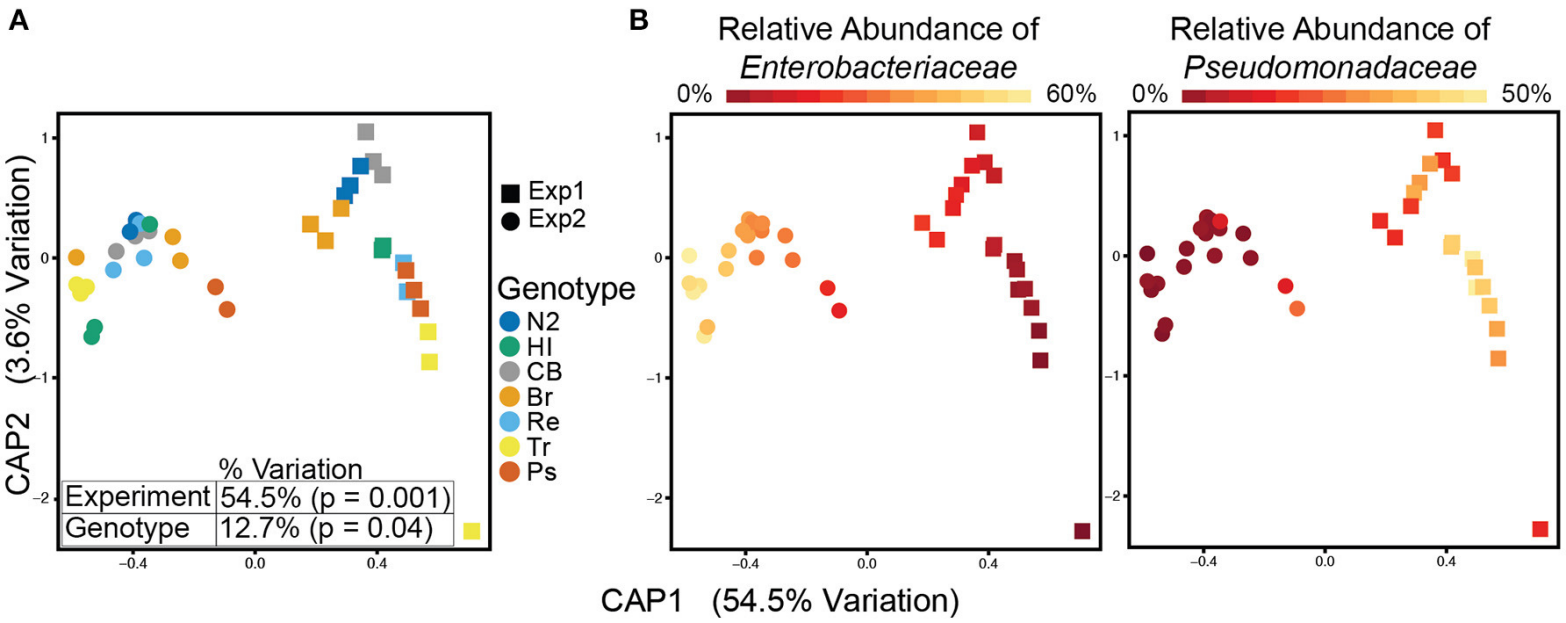
**Worm microbiota composition is affected by host genotype, but even more so by experiment-associated environmental diversity**. Shown are worm microbiotas clustered using Canonical Analysis of Principle Coordinates (CAP) based on weighted UniFrac distances. Table lists percent variation explained by the experiment or genotype variables. Significance was calculated using PERAMONVA with 1,000 permutations. Samples are colored according to host genotype and experiment **(A)**, or by relative abundance of either *Enterobacteriaceae* or *Pseudomonadaceae* **(B)**.

To substantiate the identification of genotype-specific taxa, we performed a second experiment under the same conditions as the first, but using a different soil/produce combination. In comparison to the first experiment, microbial diversity in the second soil was relatively limited (Figures S2A,B). While worm microbiotas analyzed in this experiment remained significantly distinct from their respective soil microbiota (Figures S2C,D), and formed distinct clusters for each genotype, they differed from those of the first experiment, particularly at the OTU level, and clustered away (Figure 2A and Figure S2E).

The environmental community in the second experiment was dominated by *Enterobacteriaceae*, which reflected on worm microbiotas, where members of this family made the majority, largely at the expense of *Pseudomonadaceae* (Figure S2A). Indeed, differences in the prevalence of *Enterobacteriaceae* and *Pseudomonadaceae* OTUs seemed to be an important factor in distinguishing between worm microbiotas of the two experiments (Figure 2B). To assess the contributions of experiment and host genotype to the composition of worm microbiotas, we performed a Constrained Analysis of Principal Coordinates (CAP). This showed that 54.5% of the observed variation among worm microbiotas could be explained by the experiment variable (Figure 2A). The two experiments differed in initial environmental microbial diversity, but also in the sequencing platforms used for analysis, which rely on the same chemistry but result in different read length. Results obtained with different length sequences representing the same dataset were comparable (Materials and Methods, Figure S1), indicating that differences in sequence analysis were not likely to be those responsible for differences between the two experiments, and leaving differences between environmental microbial diversity (or availability) as the likely underlying factor. While environmental diversity appeared to be a dominant factor in shaping of the worm microbiotas, host genotype also had a significant contribution, explaining 12.7% of the variation (Figure 2A). Indicator Species Analysis performed on the second experiment identified indicator OTUs that, while not overlapping with those from the first experiment, mostly belonged to the same bacterial families identified in the first, including members of the *Enterobacteriaceae* and *Pseudomonadaceae* families (Table S2 and Figure S3). Relative abundance of members of these two families varied not only between experiments, but also among the genotypes within each experiment (Figure 2B). This suggested that abundance of members of both families might be affected by host genotype.

### Functional significance of host-associated commensals

Whereas differences were apparent between microbiotas of different species or even strains, variability between experiments hindered the identification of taxa reproducibly associated with specific genotypes. This might have been exacerbated by the limited taxonomic resolution offered by 16S sequencing. To increase resolution, we turned to culture gut isolates, focusing on members of the *Enterobacteriaceae* family. *Enterobacteriaceae* were prevalent in all worm microbiotas, but differences in the abundance of some were apparent between different host genotypes; in particular, *Enterobacteriaceae* OTUs were identified as indicator species of both *C. elegans* N2 and *C. briggsae* microbiotas (Figure 1D, Tables S2, S3). We used *Enterobacteriaceae*-selective media plates to isolate gut commensals from *C. elegans* N2 and *C. briggsae* worms grown on the same soil, in two separate occasions (and with different soils). A total of 19 isolates were obtained for each species, and identified using multi-locus sequencing (MLS, Table 1). All were identified as members of the *E. cloacae* complex, or of the closely related genera *Rauoltella* and *Klebsiella*. Due to the polyphyletic nature of this clade, unequivocal identification of species is difficult even with MLS (Brady et al., 2013). Nevertheless, it appeared that even at this level of resolution, taxonomic divisions did not discriminate between the commensals of *C. elegans* and of *C. briggsae*, with both harboring a mixture of *Enterobacter, Rauoltella* and *Klebsiella* species (Table 1).

**Table 1:** ***Enterobacteriaceae* isolated from *C. elegans* and *C. briggsae***.

**Isolate^b^**	**# of isolates (% ID)**
***C. elegans*** **(N2) isolates^a^**	
*Enterobacter asburia, cloacae*, sp.	2 (100%); 4 (99%); 1 (97%)^d^
*Rauoltella ornithinolytica/Enterobacter asburiae/Klebsiella oxytoca*	1 (99%)^d^
*Rauoltella/Klebsiella/Enterobacter*	1 (98%); 1 (95%)^d^
*Citrobacter/Klebsiella/Enterobacter aerognenes, cloacae*	1 (97%)
*Cronobacter* sp.	1 (99%)
*Rauoltella ornithinolytica/Klebsiella oxytoca*	1 (100%); 2 (99%)
*Enterobacter* sp.	2 (99%)
*Enterobacter aerognenes/Citrobacter freundii*	1 (98%)
*Klebsiella oxytoca*	1 (99%)
***C. briggsae*** **isolates^a^**	
*Enterobacter asburia, cloacae*, sp.^c^	4 (100%); 3 (99%)^d^; 1 (97%)
*Citrobacter/Enterobacter/Rahnella/Yersinia*	1 (99%)^d^
*Raoultella/Klebsiella/Enterobacter*	1 (95%)
*Rauoltella ornithinolytica/Klebsiella oxytoca*	1 (100%)^d^
*Rauoltella ornithinolytica/Klebsiella oxytoca/Enterobacter aerogenes*	1 (99%)
*Enterobacter* sp.	1 (99%); 1 (98%)
*Citrobacter/Kluyvera intermedia*	1 (99%)
*Enterobacter aerogenes/Citrobacter freundii*	1 (99%)
*Raoultella ornithinolytica/Klebsiella oxytoca*	2 (99%)
*Klebsiella oxytoca*	1 (99%)

a *Identification of isolates was achieved using multi-locus sequencing, either of full-length-16S/rpoB/hsp60, or of gyrB/rpoB/hsp60; BLAST alignments were carried out with concatenated sequences*.

b *Shown are all species that received identical scores for alignment with query sequence*.

c *The group of isolates that yielded CBent1-5 (see main text)*.

d *Identified based on two available sequences*.

**Figure 3:**
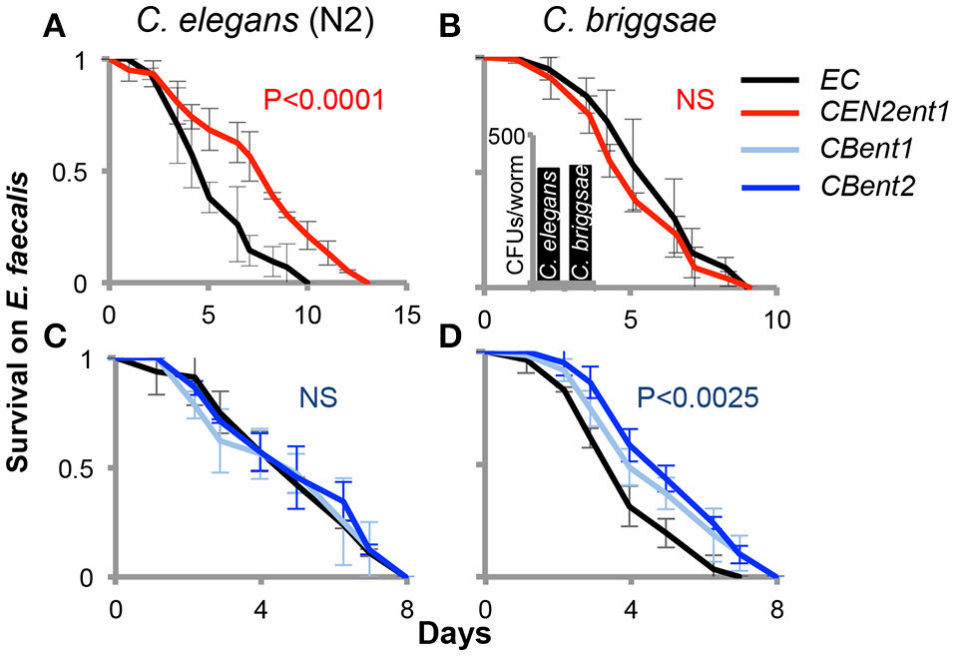
**Host-specific contributions of *C. elegans* and *C. briggsae* isolates to infection resistance**. Shown are survival curves (averages ±SDs of measurements performed in triplicate, *N* = 42–112 worms per group), for worms grown on designated isolates, and shifted at L4 to *Enterococcus faecalis*. Respective hosts are designated—*C. elegans*, in **(A,C)**, and *C. briggsae*, in **(B,D)**. Inset, *CEN2ent1* colonizes both *C. elegans* and *C. briggsae*. Shown is one experiment of two independent ones with similar trends (*N* = 129–165 worms per group). Isolates: *E*. *coli* (*EC*), *E. cloacae* isolated from *C. elegans* N2 (*CEN2ent1*), and *E. cloacae* isolated from *C. briggsae* (*CBent1, CBent2*).

Putting aside taxonomic divisions, we turned to examining the contributions of identified gut commensals to their respective host, as well as to the non-host. Previous work isolated an *E. cloacae* strain, now named *CEN2ent1*, from *C. elegans* N2 worms, and showed that it efficiently colonized worms (Berg et al., 2016). Previous work also identified a *P. mendocina* gut commensal that protected worms from infection with the pathogen *Pseudomonas aeruginosa* (Montalvo-Katz et al., 2013). We therefore examined whether the *Enterobacter* isolate was similarly capable of enhancing infection resistance. While *CEN2ent1* did not protect worms from *P. aeruginosa* infection (not shown), L4 worms raised on *CEN2ent1* showed enhanced resistance to a subsequent infection with the Gram-positive pathogen *E. faecalis* (Figure 3A). *Two* more *E. cloacae* isolates from *C. elegans* demonstrated similar capabilities. However, when tested with the non-host *C. briggsae, CEN2ent1* was incapable of conferring any protection (Figure 3B). This was in spite of equal abilities to colonize both *C. elegans* and *C. briggsae* (Figure 3 inset, Figure S5A). Following this observation of host-specific contributions, we re-examined the contribution of *P. mendocina*. This revealed a similar pattern as with *CEN2ent1*; the *P. mendocina* N2 isolate protected its *C. elegans* host from *P. aeruginosa* infection (as reported before), but did not protect *C. briggsae* (Figure S4). Interestingly, both commensals were specific to their N2 host, and did not protect two different *C. elegans* strains, the Hawaiian strain and the CB4857 strain (Figures S5B,C). We next conducted the reciprocal experiment, examining the functional significance of the *C. briggsae E. cloacae* isolates to their host, and to the non-host *C. elegans*. Five isolates were screened for their ability to increase resistance to *Enterococcus* infection, of which two were able to enhance infection resistance in *C. briggsae* (Figure 3D). However, both failed to achieve this in N2 worms (Figure 3C), corroborating the idea of host-specificity at the functional level. Together, these results indicate that specificity in host-commensal interactions may lie in the services that gut microbes provide to their hosts, even when taxonomical distinctions cannot be observed.

**Figure 4:**
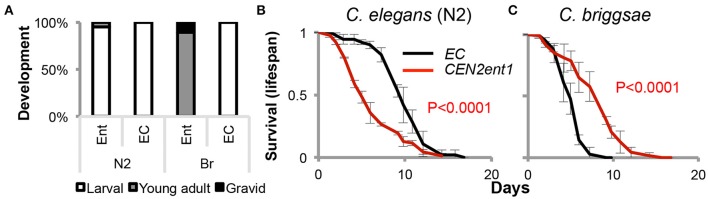
**Host-specific contributions of the *C. elegans* N2 commensal to development and lifespan. (A)** *CEN2ent1* (Ent) accelerates development of *C. elegans* N2, and even more so of *C. briggsae* (Br) compared to *E. coli* (*EC*). Developmental stage was scored 42 h after egg laying (at 25°C, *N* = 28–47 worms per group). **(B,C)** Growth on *CEN2ent1* shortens lifespan (at 25°C) of *C. elegans* worms **(B**), but not of *C. briggsae* **(C)**. Averages ±SDs of measurements performed in triplicate (*N* = 31–89 worms per group).

Differential effects of the commensals on host versus non-host were also observed beyond infection resistance. *CEN2ent1* accelerated the development of both its *C. elegans* host and the non-host *C. briggsae*, but acceleration was much more prominent in the non-host (Figure 4A, Figure S6A, and results not shown). No significant effects were observed on fecundity of either host species, but *C. elegans* worms grown continuously on *CEN2ent1* presented a significantly shortened lifespan (Figure 4B). These effects were observed in all three examined *C. elegans* species (Figure S6B). In contrast, *C. briggsae* grown on *CEN2ent1* lived longer than their siblings grown on *E. coli* (Figure 4C). Thus, the *C. elegans* commensal affects various life history traits of its host, particularly during development and early adulthood. Importantly, these effects are host-specific and may be very different in worms that are not the original host.

## Discussion

Our results allow gauging of the relative contributions of environmental diversity and host genetics to shaping of the gut microbiota, demonstrating contributions of both, with a greater contribution for the environment. Regardless of the experiment and the environmental microbial diversity, worms (specifically *C. elegans* and *C. briggsae*) reproducibly harbored bacteria of the *E. cloacae* clade. The resolution offered by 16S rDNA sequencing, or even by multi-locus sequencing of isolates, could not discriminate between members of this group, and hindered identification of microbial species specific to particular host genotypes. However, functional evaluation of the commensals revealed specificity, with several *C. elegans* N2 isolates providing infection protection to their host, but not to the non-host *C. briggsae*, or even to other *C. elegans* strains, and *C. briggsae* isolates providing infection protection to their own host, but not to *C. elegans*.

Our results indicate contributions of both host genetics and environment to shaping of the microbiota. Whereas, environmental diversity in the different experiments strongly affected gut microbiota composition, co-clustering of microbiotas by genotype was preserved (Figure 2A), suggesting that effects of the environment were modulated by host genotype. This dual contribution could be the result of two different mechanisms. In one, gene-environment interactions could have combined effects on the abundance of all microbiota constituents; alternatively, host genetics and environmental diversity could have non-overlapping contributions affecting the abundance of distinct taxa. Additional experiments will be required to differentiate between the two possibilities.

The hologenome concept provides a model that incorporates contributions of both host genetics and environmental diversity to shaping of gut microbiotas. Considering the host genome and the genomes of its microbes as one unit under selection, this model suggests selectable interactions between host and commensal genes, but also considers the advantage of exchanging microbes with the environment as a way to increase hologenetic variation (Zilber-Rosenberg and Rosenberg, 2008; Bordenstein and Theis, 2015; Shapira, 2016). The relative prevalence of host-specific commensals versus variant environment-dependent microbes would dictate the relative contributions of host genetics and environmental diversity to gut community composition, and may vary according to the natural history of the host. Considering that of *Caenorhabditis* nematodes, it might be expected that environmental diversity would play a dominant role, due to the continuous exposure of worms to unstable soil habitats, and due to the bacteriovoric lifestyle of most of them.

Overall, examined host species harbored microbiotas that were quite similar, with high prevalence of *Enterobacteriaceae, Pseudomonadacae*, and *Xanthomonadacae*, in agreement with previous reports (Berg et al., 2016; Dirksen et al., 2016). This suggests general constraints imposed by the intestine niche on microbiota structure that are shared by all nematodes. Interestingly, Ps, a parasitic worm of insects serving as an outlier in this study, also harbored a similar microbiota. This is in agreement with previous reports identifying members of the families described here in *Pristionchus* raised in soil or in beetles (Rae et al., 2008).

Beyond gross similarities, host-specific differences in microbiota composition were observed in sequence data, but were not reproducible at the OTU level between experiments. This is not completely surprising considering that soil microbial composition between the two experiments (i.e., the worm inoculum) was very different, and would understandably give rise to differences at the OTU level. Nevertheless, this hindered high-confidence identification of host-specific taxa. Overcoming this, functional analysis was able to identify host-specific commensals based on services that they provided only to their host. Protection from *Enterococcus* infection is one of them. This is likely a significant benefit in the wild, as pathogens of this family are found in soil, posing a threat to worms (Staley et al., 2014), and thanks to the dilution-insensitivity of the commensal's protective effects (Figure S7A). However, the N2 commensal could also be disadvantageous to its host, by shortening lifespan. While the specificity of this phenotype to *C. elegans* strains further demonstrates a genotype-specific contribution, this detrimental phenotype is lost in a mixed culture (Figure S7B), suggesting that it is likely not playing a significant role under natural circumstances.

Our results demonstrate host-specificity within the gut microbiota that is overlooked by phylogenetic analyses, but can be revealed by functional evaluation. A similar result was reported in mice, where proper development of the immune system in germ-free animals depended on gut microbiota transplanted from normal mice, and was defective when transplants were made from humans, or even from rats, in spite of an overall similarity between the microbiotas of the different host species (Chung et al., 2012). Since most evaluations of host genetics as a factor shaping the gut microbiota are carried out through sequencing, it is possible that this role is systematically underestimated. The resolution offered by deep sequencing could be improved, as described in a recent study, relying on increased diversity within the bacterial *gyrB* gene family. The improved resolution enabled unraveling co-speciation of gut commensals with their hominid hosts, supporting the roles of host genetics and of evolution in shaping microbiota composition (Moeller et al., 2016). However, these roles might be even greater, as suggested by the functional host-specificity that we identified, which could not be discerned even by *gyrB* sequencing.

## Author contributions

MB, XZ, and MS contributed to the preparation of the manuscript. Experiments and data analyses were carried out by MB and XZ.

### Conflict of interest statement

The authors declare that the research was conducted in the absence of any commercial or financial relationships that could be construed as a potential conflict of interest. The reviewer DC declared a shared affiliation, though no other collaboration, with the authors to the handling Editor, who ensured that the process nevertheless met the standards of a fair and objective review.
