# Supplementary material for: Host-Specific Functional Significance of Caenorhabditis Gut Commensals
Source: Front Microbiol. 2016 Oct 17;7:1622. doi: 10.3389/fmicb.2016.01622 (PMC5066524; doi:10.3389/fmicb.2016.01622)
Supplement: Supplementary file 2 [file DataSheet2.docx]

**Table S2. Indicator taxa shared between experiments.**

| **Genotype** | **Analysis of single-end reads^a^** | **Of paired-end reads** |
| --- | --- | --- |
| *C. elegans*,  strain N2 | ***Enterobacteriaceae^b^ Paenibacillaceae***  ***Rhizobiaceae Sphingomonadaceae*** | ***Enterobacteriaceae Paenibacillaceae***  ***Rhizobiaceae Sphingomonadaceae*** |
| *C. elegans*, Hawaiian strain | ***Enterobacteriaeae*** | ***Enterobacteriaeae*** |
| *C. elegans*,  strain CB4857^c^ | ***Bacillaceae***  *Microbacteriaceae Rhodobacteraceae* | ***Bacillaceae***  *Rhizobiaceae Sphingomonadaceae Sphingobacteriaceae* |
| *C. briggsae* | ***Comamonadaceae*** | ***Comamonadaceae*** *Enterobacteriaeae* |
| *C. remanei* | *Xanthomonadaceae* | *Sphingomonadaceae* |
| *C. tropicalis* | none | none |
| *Pristionchus pacificus* | ***Xanthomonadaceae*** ***Paenibacillaceae Cytophagaceae Pseudomonadaceae*** | ***Xanthomonadaceae Paenibacillaceae Cytophagaceae Pseudomonadaceae*** *Bacillaceae Propionibacteriaceae* |

^a^ Overlap between experiment 1 and 2 is shown for families of Indicator species, identified at the OTU level for each experiment separately, and pooled at the family level. Shown are families, representatives of which were identified in both experiments (only marginal overlap was observed at the OTU level). In experiment 2, indicator analysis was performed using either data based on the forward read of the MiSeq paired-end sequencing library (single reads), or data based on longer sequences generated by the overlapping paired-end forward and reverse reads from the same library. Overlap between indicators identified by the two types of analysis was almost complete, with unique families represented only by rare OTUs. A full list of all indicator taxa and their prevalence can be found in Table S3.

^b^ Families shown in bold are those identified in all analyses.

^c^ Unlike indicators of other genotypes, those of *C. elegans* strain CB4857 were not enriched, but rather depleted in CB4857 worms compared to soil (see Fig. 1D).
