# Supplementary material for: Host-Specific Functional Significance of Caenorhabditis Gut Commensals
Source: Front Microbiol. 2016 Oct 17;7:1622. doi: 10.3389/fmicb.2016.01622 (PMC5066524; doi:10.3389/fmicb.2016.01622)
Supplement: Supplementary file 4 [file Image1.PDF]

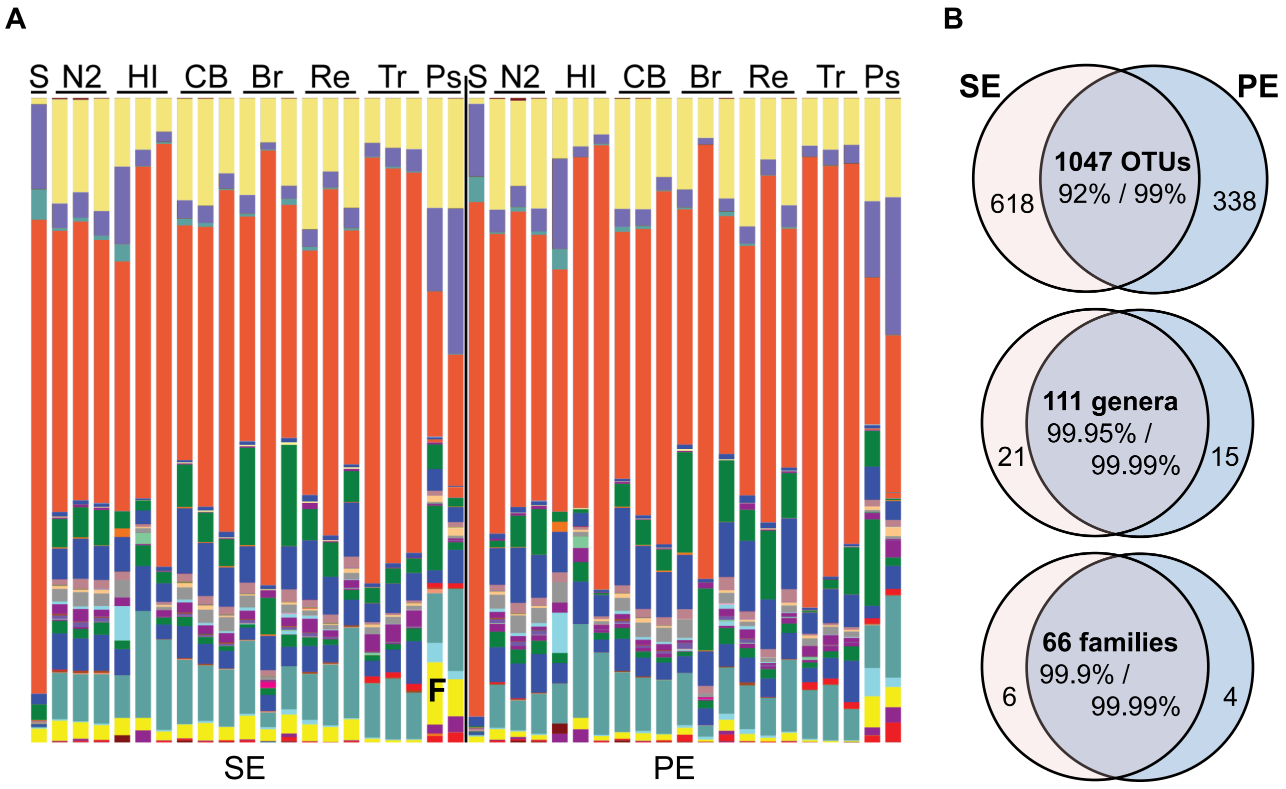

Figure S1. Similar microbiota composition is inferred by analysis based on single-end or paired-end reads of the same library. (A) Soil and worm microbiota composition in samples of experiment 2, inferred based on 250 bp forward reads of paired-end 16S sequencing libraries (SE), or on 350 bp composite reads assembled from forward and reverse paired-end reads (PE) of the same libraries. Each bar represents a microbiota from a worm population, or from their soil environment (S), showing relative abundance of taxa (family-level, color-labeled). Included are *C. elegans* strains N2, Hawaiian (HI), and CB4857 (CB), *C. briggsae* (Br), *C. remanei* (Re), *C. tropicalis* (Tr), and *Pristionchus pacificus* (Ps). *Flavobacteriaceae* (F) was one of the few taxa that demonstrated a significant change in relative abundance with PE sequence analysis. **(B) The great majority of OTUs, genera, and families were detected using either single-end or paired-end sequencing.** Venn diagrams show numbers of taxa, at the designated levels, overlapping between SE and PE analyses (center), or unique, and percent overlap for reads associated with the shared taxa.
