# Supplementary material for: Host-Specific Functional Significance of Caenorhabditis Gut Commensals
Source: Front Microbiol. 2016 Oct 17;7:1622. doi: 10.3389/fmicb.2016.01622 (PMC5066524; doi:10.3389/fmicb.2016.01622)
Supplement: Supplementary file 5 [file Image2.PDF]

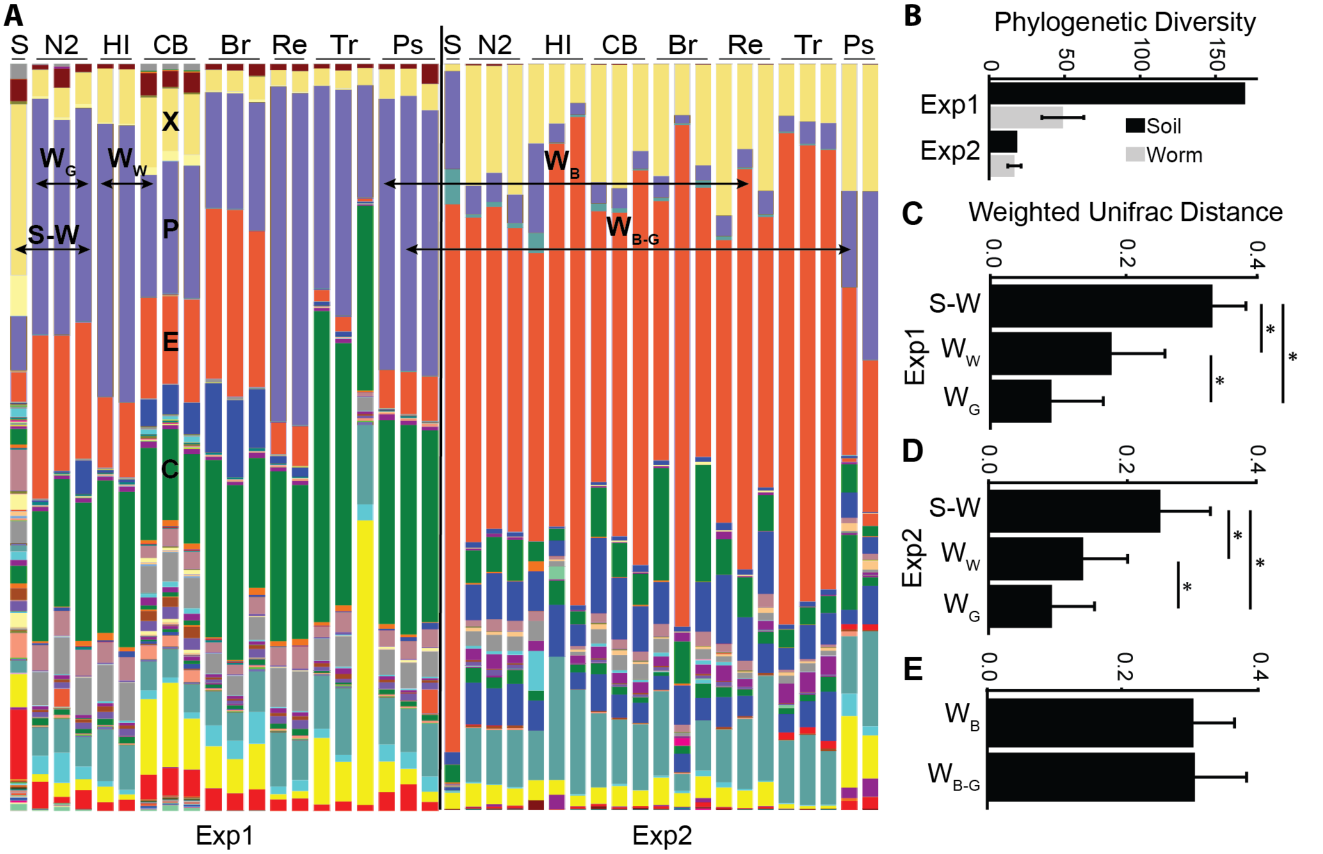


**Figure S2. Genotype-associated similarities in worm microbiota composition are reproducible, but diluted by differences associated with independent experiments. (A)** Soil and worm microbiota composition. Each bar represents a microbiota from a worm population (>100 worms), or from their soil environment (S)(1 gr), showing relative abundance of taxa (family-level, color-labeled). Two independent experiments are shown. Worms include *C. elegans* strains N2, Hawaiian (HI), and CB4857 (CB), *C. briggsae* (Br), *C. remanei* (Re), *C. tropicalis* (Tr), and *Pristionchus pacificus* (Ps). Highlighted major families include *Enterobacteriaceae* (E), *Xanthomonadaceae* (X), *Pseudomonadaceae* (P), and *Comamonadaceae* (C). **(B)** Microbial diversity in soil versus averages ± SDs for 19 worm microbiotas in Exp1 and 20 worm microbiotas in Exp2. **(C,D)** Weighted distances between microbiotas in either Exp1 **(C)** or Exp2 **(D)** demonstrate greater similarity in worms of the same genotype ($W_G$), than in worms of different genotypes ($W_W$), and further greater than similarities between worm microbiotas and the soil microbiota (S-W); averages ± SDs for all possible pair-wise comparisons; *, $p < 0.001$ (Student's t-test with 1,000 Monte Carlo permutations). **(E)** Comparisons of weighted distances between worm microbiotas of Exp1 and Exp2, either within the same genotype ($W_{B-G}$) or all vs. all ($W_B$), demonstrate dilution of discernable genotype-associated effects.
