# Supplementary material for: Host-Specific Functional Significance of Caenorhabditis Gut Commensals
Source: Front Microbiol. 2016 Oct 17;7:1622. doi: 10.3389/fmicb.2016.01622 (PMC5066524; doi:10.3389/fmicb.2016.01622)
Supplement: Supplementary file 6 [file Image3.PDF]

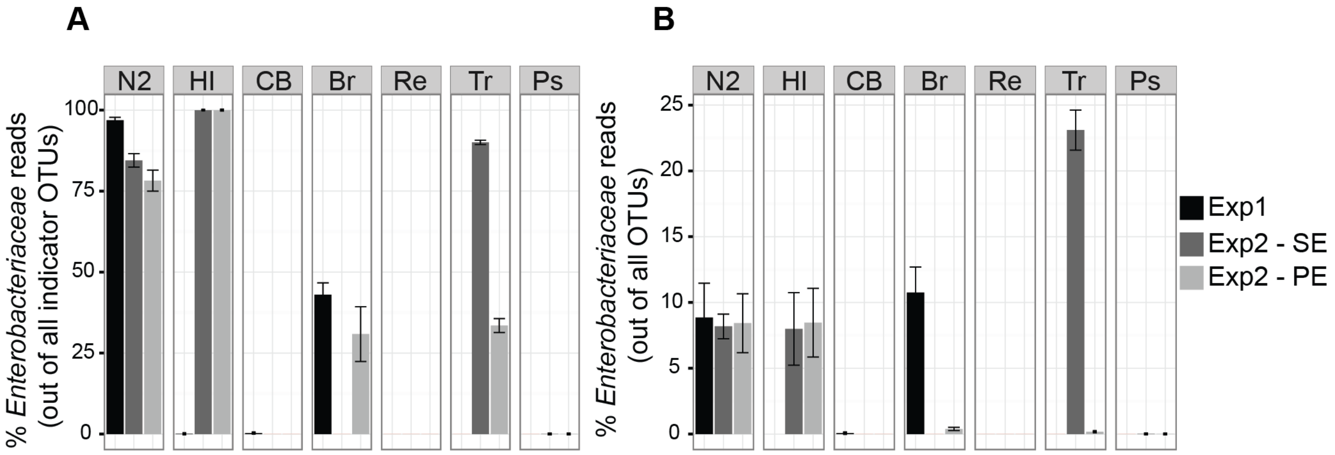

Figure S3. *Enterobacteriaceae* species are commonly identified as indicator taxa distinguishing between microbiotas of different worm genotypes. (A) *Enterobacteriaceae* make a high portion of identified indicator OTUs in both experiments. Shown are percentages of reads associated with *Enterobacteriaceae* out of the different genotype-specific indicator OTU reads, in both experiments 1 and 2 (designated), and as revealed with different types of sequence analyses (of experiment 2 data, either using single-end (SE) sequencing, or paired-end (PE) sequencing). Averages ± SDs for 2-3 independent populations for each genotype, and for each experiment. **(B) Reads associated with indicator *Enterobacteriaceae* species make a relatively large portion of the total number of reads for each microbiota**. Values as in A, with percentages representing *Enterobacteriaceae* OTU reads out of all OTU reads per genotype. Genotype designations are as in Fig. 1.
