# Supplementary material for: Host-Specific Functional Significance of Caenorhabditis Gut Commensals
Source: Front Microbiol. 2016 Oct 17;7:1622. doi: 10.3389/fmicb.2016.01622 (PMC5066524; doi:10.3389/fmicb.2016.01622)
Supplement: Supplementary file 7 [file Image4.PDF]

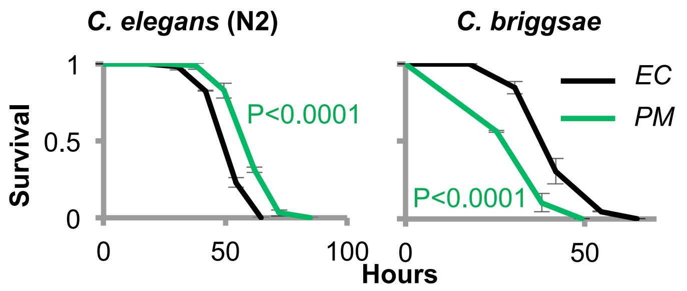

Figure S4. Growth on *Pseudomonas mendocina* (*PM*), an N2 isolate (Montalvo-Katz, et al., 2013) confers resistance to subsequent *Pseudomonas aeruginosa* infection in N2 worms but not in C. briggsae, the latter further sensitized to infection by growth on this N2 commensal. Shown are averages ± SDs of measurements performed in triplicate (N=59-164 worms per group). Control worms were grown on *E. coli* (*EC*).
