# Supplementary material for: Host-Specific Functional Significance of Caenorhabditis Gut Commensals
Source: Front Microbiol. 2016 Oct 17;7:1622. doi: 10.3389/fmicb.2016.01622 (PMC5066524; doi:10.3389/fmicb.2016.01622)
Supplement: Supplementary file 8 [file Image5.PDF]

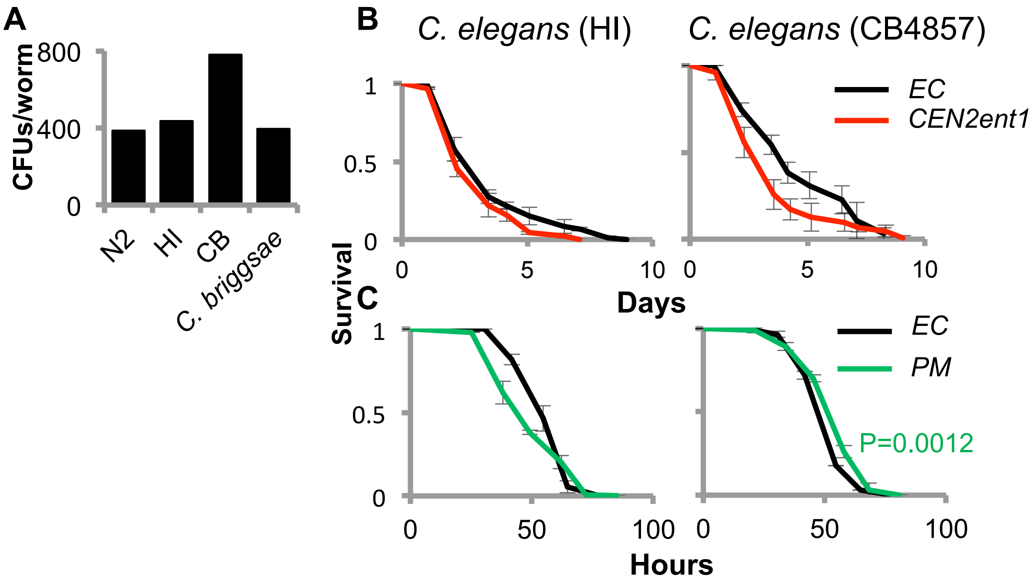

Figure S5. N2 commensals are specific to their host to the extent that they cannot protect other *C. elegans* strains from infection. (A) *CEN2ent1* also colonizes additional strains of *C. elegans,* such as the Hawaiian strain (HI) and CB4857 (CB). Shown is one experiment of two independent ones with similar results (N=77-165 worms per group). **(B,C)** N2 commensals do not (or only marginally) protect other *C. elegans* strains from infection. Shown are survival curves from worms raised on *CEN2ent1* and subsequently exposed to the pathogen *Enterococcus faecalis* **(B)**, or worms raised on *Pseudomonas mendocnia*, and subsequently exposed to the *P. aeruginosa* pathogen **(C)**. Averages ± SDs of measurements performed in triplicate (N=92-189 per group). Isolates: *E. coli* (*EC*), *E. cloacae* isolated from *C. elegans* N2 (*CEN2ent1*), and *P. mendocina* isolated from N2 *C. elegans* (*PM*).
