# Supplementary material for: Host-Specific Functional Significance of Caenorhabditis Gut Commensals
Source: Front Microbiol. 2016 Oct 17;7:1622. doi: 10.3389/fmicb.2016.01622 (PMC5066524; doi:10.3389/fmicb.2016.01622)
Supplement: Supplementary file 9 [file Image6.PDF]

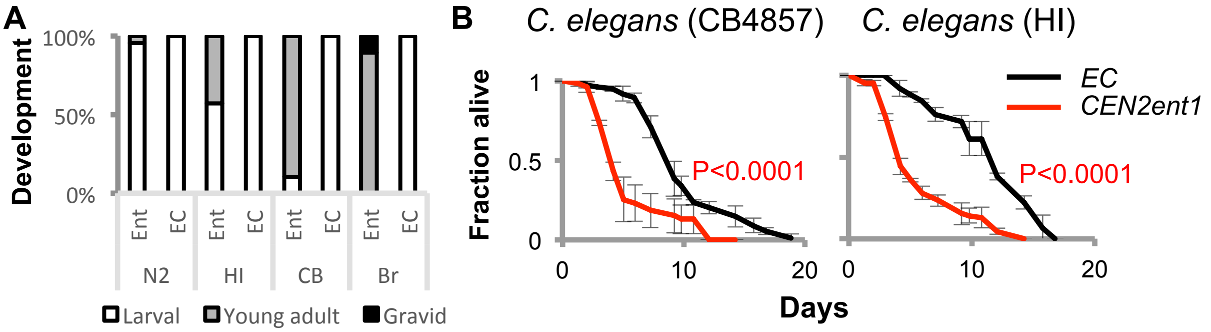

Figure S6. Effects of the *C. elegans* N2 commensal on development and lifespan of different *C. elegans* strains. (A) *CEN2ent1* (Ent) accelerates development of *C. elegans* N2, and even more so of the *C. elegans* Hawaiian (HI), and CB4857 (CB) strains, and of *C. briggsae* (Br) compared to *E. coli* (*EC*). Developmental stage was scored 42 hours after egg laying (at 25ºC, N=28-47 worms per group). **(B)** Growth on *CEN2ent1* shortens lifespan (at 25ºC) of all *C. elegans* strains. Averages ± SDs of measurements performed in triplicate (N=66-86 worms per group).
