# Supplementary material for: Host-Specific Functional Significance of Caenorhabditis Gut Commensals
Source: Front Microbiol. 2016 Oct 17;7:1622. doi: 10.3389/fmicb.2016.01622 (PMC5066524; doi:10.3389/fmicb.2016.01622)
Supplement: Supplementary file 10 [file Image7.PDF]

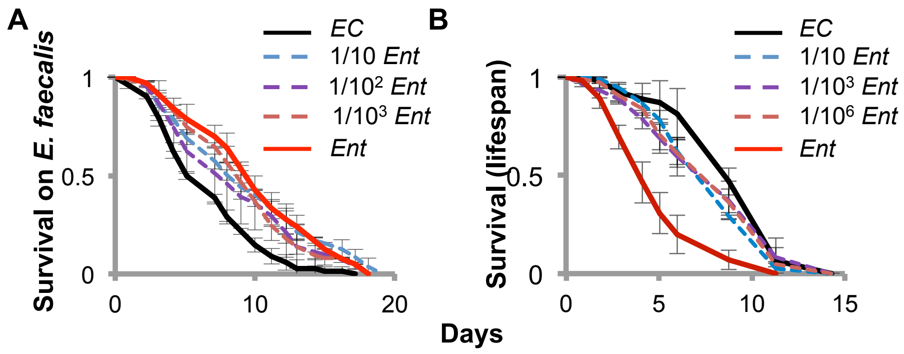

Figure S7. Infection protection provided by the N2 commensal is insensitive to dilution, but not effects of th ecommensal on lifespan. (A) Protection provided by *E. cloacae* (*CEN2ent1*, *Ent* in short) from a subsequent infection with *E. faecalis* is insensitive to dilution. Averages ± SDs of measurements performed in duplicate (N=72-96 L4 worms per group; $p<0.05$ for all comparisons to worms initially grown on *E. coli* (*EC*). **(B)** Lifespan shortening by growth on *CEN2ent1* disappears upon dilution with *E. coli* (N=104-113 worms per group).
